# Supplementary material for: Splice-Junction-Based Mapping of Alternative Isoforms in the Human Proteome
Source: Cell Rep. Author manuscript; Available in PMC 2020 Jan 15. (PMC6961840; doi:10.1016/j.celrep.2019.11.026)
Supplement: 2 [file NIHMS1546469-supplement-2.zip › DF1/PXD006675/VentricularSeptum/VentricularSeptum_36_SYPL1_ETSLHSPSNTSAPHSQGGIPPPTGL.pdf]

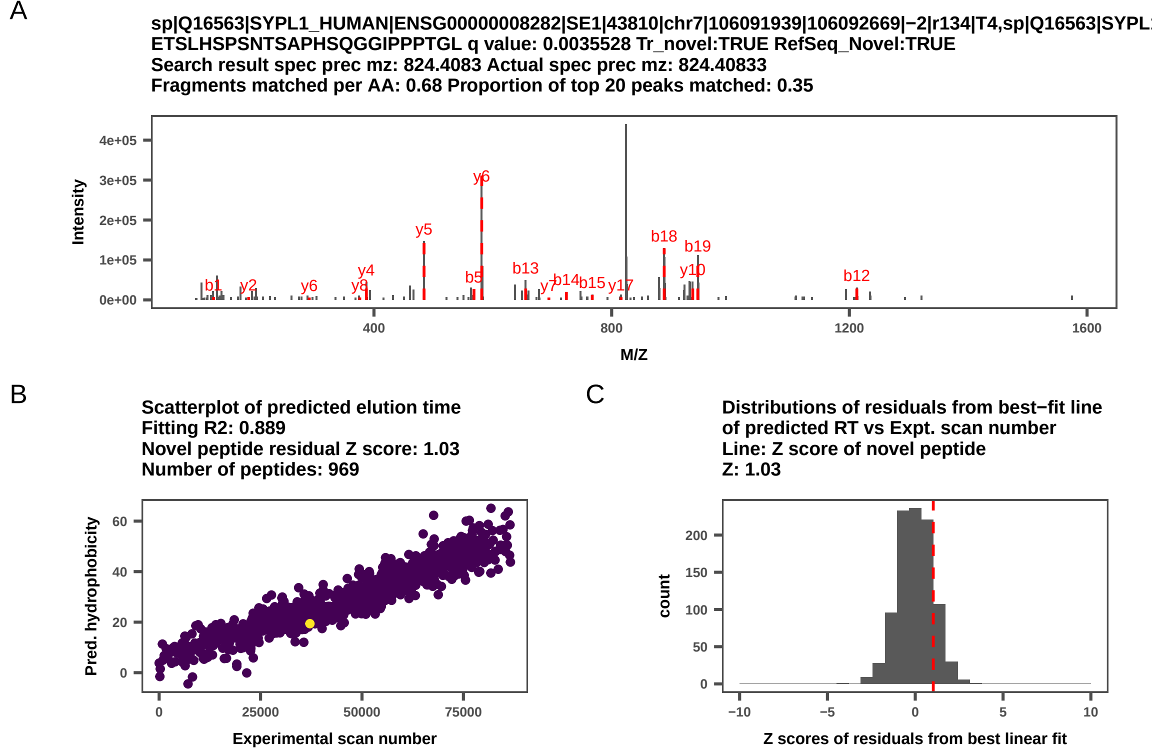
A
sp|Q16563|SYPL1_HUMAN|ENSG00000008282|SE1|43810|chr7|106091939|106092669|−2|r134|T4,sp|Q16563|SYPL
ETSLHSPSNTSAPHSQGGIPPPTGL q value: 0.0035528 Tr_novel:TRUE RefSeq_Novel:TRUE
Search result spec prec mz: 824.4083 Actual spec prec mz: 824.40833
Fragments matched per AA: 0.68 Proportion of top 20 peaks matched: 0.35
Intensity
M/Z
B
Scatterplot of predicted elution time
Fitting R2: 0.889
Novel peptide residual Z score: 1.03
Number of peptides: 969
Pred. hydrophobicity
Experimental scan number
C
Distributions of residuals from best−fit line
of predicted RT vs Expt. scan number
Line: Z score of novel peptide
Z: 1.03
count
Z scores of residuals from best linear fit
